# Isotope Effects on Chemical Shifts in the Study of Intramolecular Hydrogen Bonds

**DOI:** 10.3390/molecules20022405

**Published:** 2015-01-30

**Authors:** Poul Erik Hansen

**Affiliations:** Department of Science, Systems and Models, Roskilde University, Universitetsvej 1, Roskilde DK-4000, Denmark; E-Mail: poulerik@ruc.dk; Tel.: +45-4674-2432; Fax: +45-4674-3011

**Keywords:** RAHB, isotope effects on chemical shifts, tautomeric systems

## Abstract

The paper deals with the use of isotope effects on chemical shifts in characterizing intramolecular hydrogen bonds. Both so-called resonance-assisted (RAHB) and non-RAHB systems are treated. The importance of RAHB will be discussed. Another very important issue is the borderline between “static” and tautomeric systems. Isotope effects on chemical shifts are particularly useful in such studies. All kinds of intramolecular hydrogen bonded systems will be treated, typical hydrogen bond donors: OH, NH, SH and NH^+^, typical acceptors C=O, C=N, C=S C=N^−^. The paper will be deal with both secondary and primary isotope effects on chemical shifts. These two types of isotope effects monitor the same hydrogen bond, but from different angles.

## 1. Introduction

**Figure 1:**
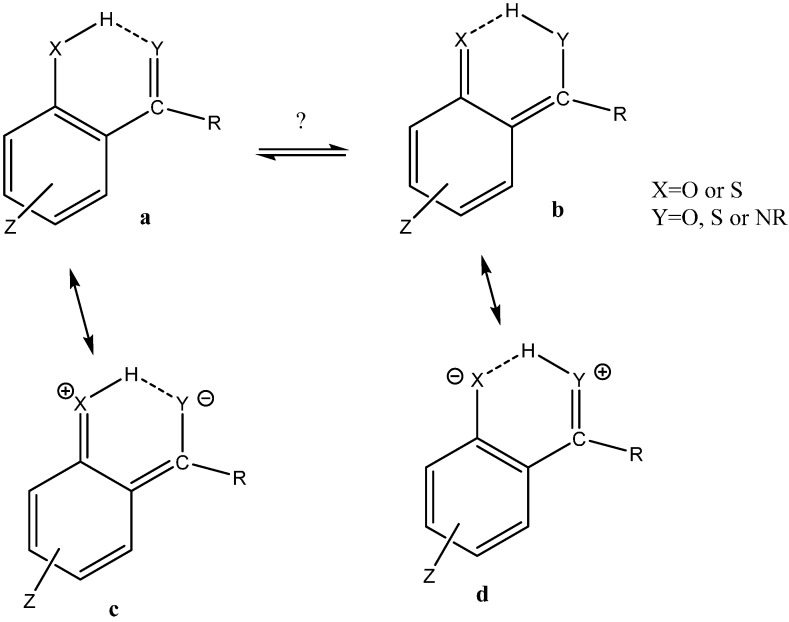
Resonance and tautomerism.

**Figure 2:**
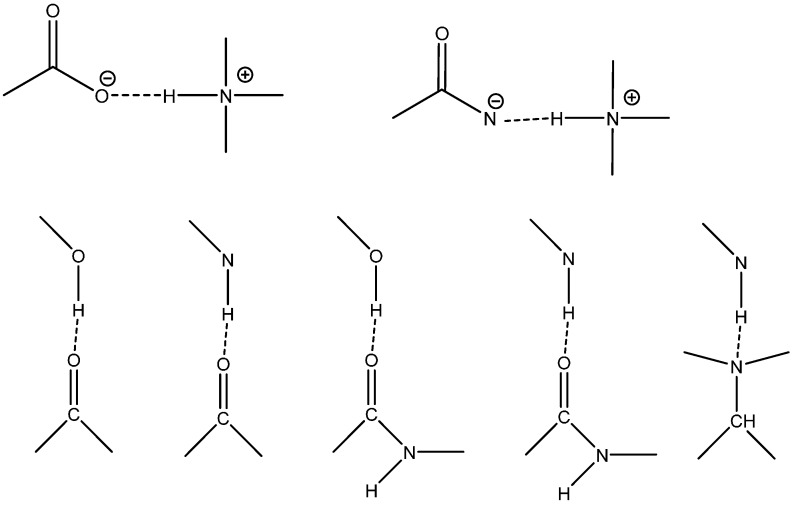
Hydrogen bond motifs.

The term intramolecular means within a molecule but even within molecules the type of hydrogen bonds can be different. A very important distinction is between hydrogen bonds that have been termed RAHB and those not (see Figure 1). If not RAHB the intramolecular hydrogen bonds may be very similar to intermolecular hydrogen bonds (examples are hydrogen bonds typically found in proteins). Examples are given in Figure 2.

**Figure 3:**
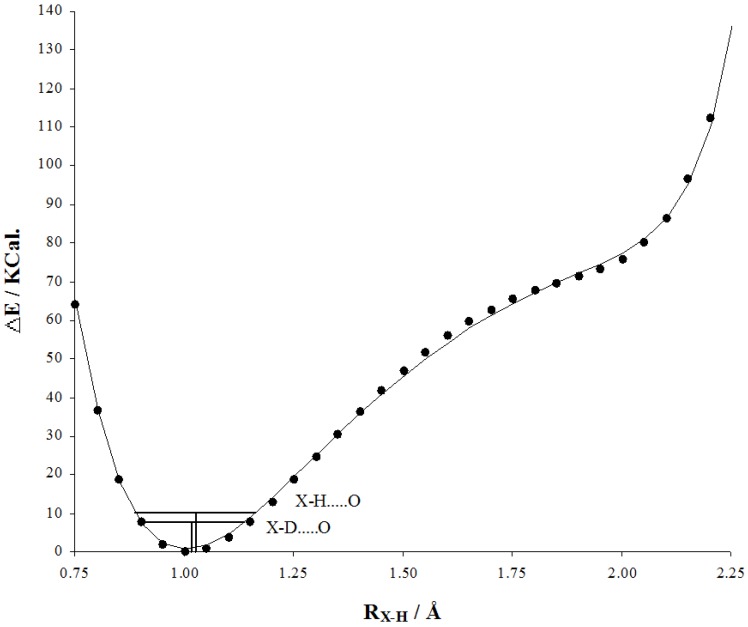
Calculated hydrogen bond potential of the formamide dimer (MP2/6-311G(d,p).

^n^ΔX(D)_int_ = (1 − χ_D_) ^n^ΔX(D)_OH_ + χ_D_^n^X(D)_NH_ (1)

^n^ΔX(D)_eq_ = (δX_NH_ − δX_OH_) Δχ (2)

^n^ΔX(D)_OBS_ = ^n^ΔX(D)_int_ + ^n^ΔX(D)_eq_ (3)

The use of isotope effects on chemical shifts (IECS) has been described in many reviews [1,2,3,4]. Both primary and secondary isotope effects on chemical shifts will be discussed. IECS are very useful in the study of hydrogen bonding as the effect is caused by variations in the zero point energy and therefore strongly related to the shape of the hydrogen bond potential as seen in Figure 3. This should ideally be a multi surface potential [5]. IECS are in the present paper defined as: ^n^ΔX(D) = δX(H) − δX(D) exemplified for deuterium isotope effects; n is the number of bonds between the deuterium and the nucleus in question. The definition for primary isotope effects is: ^P^Δ(D) = δ(H) − δ(D) [6]. Unfortunately the opposite is also recommended [7]. The definition in Equation (1) is used in the present text and all numbers given are according to this definition. With respect to naming the isotope effects different nomenclatures are used. One way is to refer to the isotope effects as intrinsic. This goes back to Jameson [8]. For symmetrical equilibrium systems in which isotope substitution does not lift the redundancy may still be referred as intrinsic as deuteriation is not perturbing the equilibrium. For non-symmetrical systems an equilibrium part will also occur (see equilibrium isotope effects). As the intrinsic isotope effect is caused by a change in the average bond length upon deuteriation (Figure 3) the effect is also referred to as geometric [9]. The intrinsic isotope effects (see Definition) and the equilibrium contribution may be separated leading to a simple approach:

In Equations (1)–(3) χ_D_ is the mole fraction, Δχ, the change in the molefraction upon deuteriation, ^n^ΔX(D) is the isotope effect for nucleus X due to deuteriation n bonds away. Int, eq and OBS refer to intrinsic, equilibrium and observed. 

The above description is based on the Born-Oppenheimer approximation as described by Jameson [8]. The observation of a change in the solid in the heavy atom distance upon deuteriation, the so-called Ubbelohde effect [10] indicates that deuteriation may influence the geometry. However, no Ubbelohde effects have been reported in intramolecular hydrogen bonded systems [1]. For intermolecular hydrogen bonded systems this is certainly the case [9], so for the intramolecular cases resembling intermolecular hydrogen bonding this should be taken into account.

The intramolecular cases can be formulated in a different way see Limbach *et al.* [11]. A thorough discussion of the difference between the two approaches is found in Ref. [12]. For intramolecularly hydrogen bonded cases involving 2,6-dihydroxy acyl aromatics also entropy has to be taken into account [13]. Another highly connected issue is tautomerism. However, this has been treated in some detail lately [3,4]. This review will concentrate on recent developments.

## 2. Results and Discussion

### 2.1. Isotope Effects of RAHB Cases

The term RAHB was first coined by Gilli *et al.* RAHB and they analyzed in great detail a very large number of examples mainly based on X-ray results [14,15]. The essence of the RAHB system is a donor, an acceptor and a bond with double bond character joining the two and of course the charge generation in the resonance form (see Figure 1d). The importance of the concept has since been disputed by Alkorta *et al.* [16,17,18].

#### 2.1.1. Secundary Isotope Effects

**Figure 4:**
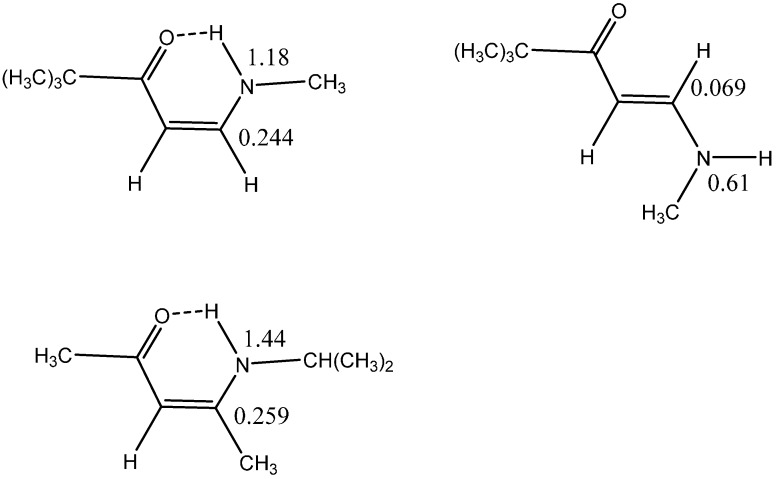
Enaminones (isotope effects in ppm).

As shown early on in enaminones, deuterium isotope effects on chemical shifts, ^13^C or ^15^N can monitor intramolecular hydrogen bonding very well as shown in Figure 4. It is obvious that the IECS over one bond at ^15^N (1.18 or 1.44 *vs.* 0.61 ppm) or over two bonds at ^13^C (0.244 or 0.259 *vs.* 0.069 ppm) are much larger in the hydrogen bonded cases.

Principal component analysis of a number of *o*-hydroxy acyl nitro aromatics (Figure 1, Z=NO_2_) showed a relationship between two-bond isotope effects on ^13^C chemical shifts and the bond lengths around the hydrogen bond system in agreement with resonance assisted behavior [19]. The characteristics of a RAHB pattern is that the O…O, the O…H, the C-OH and the C-C=O distances become shorter with stronger hydrogen bonds, whereas the O-H, and C=O bond lengths become longer. Furthermore, the negative charge at the OH oxygen is supposed to go down whereas that at the C=O oxygen is supposed to increase. For *o*-hydroxyesters, covering a very wide range of compounds, we find exactly the same type of coefficient pattern analyzing the two-bond isotope effects *vs.* bond lengths [20].

**Figure 5:**
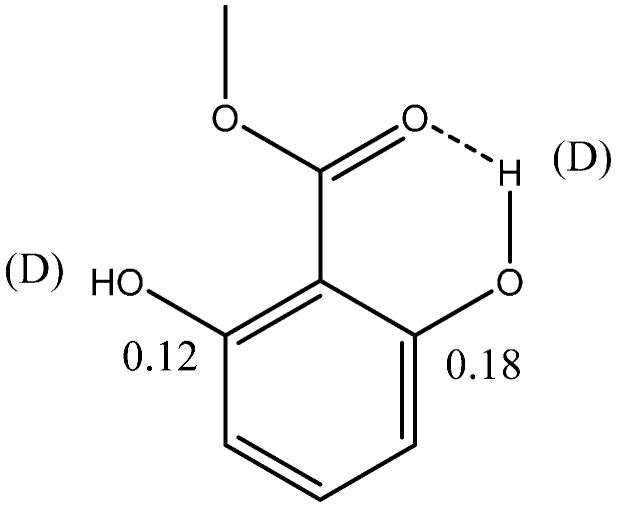
Isotope effects (in ppm) in methyl 2,6-dihydroxybenzoate. Data from Ref. [21].

A classic case is that of methyl 2,6-dihydroxybenzoate (Figure 5), which at low temperature shows two different OH chemical shifts (11.8 and 8.6 ppm) and two different two-bond deuterium isotope effects at C-2 and C-6 (0.18 and 0.12 ppm) indicating that the two hydrogen bonds are clearly different despite the fact that the O…O distances only differ by 0.02 Å. The one to C-2 is in a RAHB systems, whereas the one to C-6 is not. See also below for a discussion of the importance of the O…O distance.

**Figure 6:**
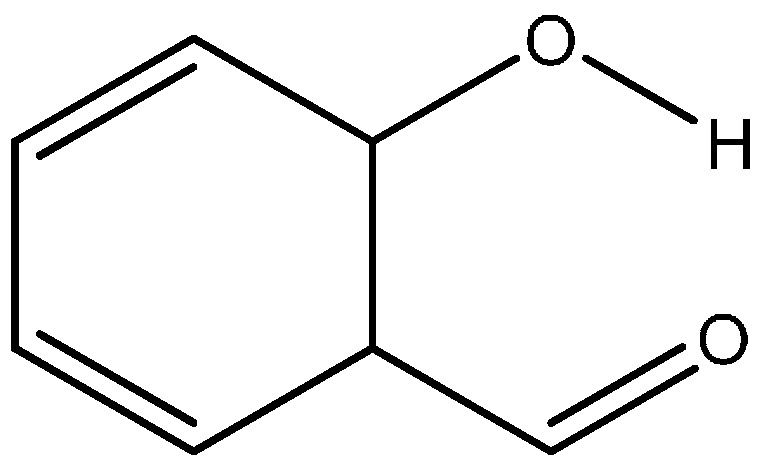
Example of a molecule transformed from salicylaldehyde but with a single bond between the acceptor and the donor.

Sanz *et al.* claim that RAHB is not important but only the oxygen-oxygen distance in the intramolecular hydrogen bond is of importance. They support their idea by calculating a number of intramolecular hydrogen bonds created artificially created by changing a double bond into a single bond and keeping the atoms in the plane [16]. An example is as seen in Figure 6. This of course is an energetically very costly manner, but by looking at electron densities at bond critical points *etc.* they claim to have strong hydrogen bonds. One point of criticism is that the methods they use are taken from intermolecular hydrogen bonds. Furthermore, nature is not creating such strong hydrogen bonds with single bonds joining the donor and the acceptor atoms.

In Table 1 are given a couple of data illustrating that the heavy atom distance is not the determining factor.

**Figure 7:**
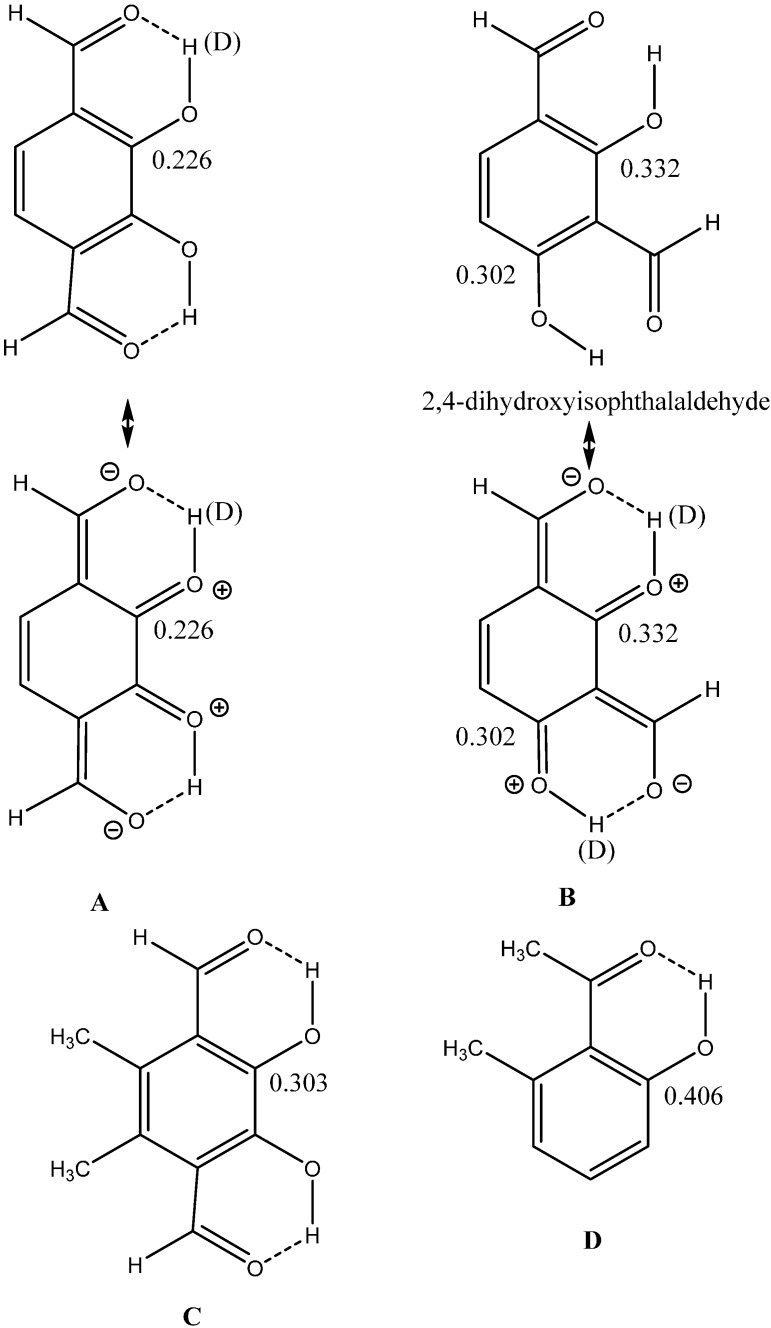
Aldehydes. Data for D taken from Ref. [26].

Another example of RAHB is the aldehydes shown in Figure 7. The two-bond isotope effects (shown in many cases to be good monitors of hydrogen bond strength [1,2,3,4,5]) are clearly different in the 2,3-dihydroxyterephthalaldehyde (**2,3-dt**) and in 2,4-dihydroxyisophthalaldehyde (**2,4-di**). In the former positive charge is repulsive in the charge separated form and the isotope effect is clearly smaller than in the latter. The distances around the hydrogen bond systems are given in Table 2.

**Table 1:** Calculated heavy atom distances and two-bond deuterium isotope effects on ^13^C chemical shifts.

Compound	R_N…X_ in Å ^a^	^2^ΔC(ND) in ppm	Refs.
2,4-dinitro-N,N-(naphthalene-1,8-diyl)bis(2,2,2-trifluoracetamide) See Figure 13	2.5968	0.32	[22]
1,4-diaminoanthraquinone	2.5564	0.36	[23]
1,4-diphenylaminoanthraquinone	2.5791	0.28	[23]
(Z)-N-methyl-3-phenyl-1-amino-3-propa-1-enone	2.6552	0.24	[24]
(Z)-N-phethyl-3-phenyl-1-amino-3-propa-1-enone	2.6556	0.302	[24]

^a^ Calculated using the Gaussian program [25] in the B3LYP functional and the 6-31G(d,p) basis set.

**Table 2:** Distances around the hydrogen bond in Å for dihydroxyterephthalaldehydes.

Compound	C=O	C-C	C=C	C-O	O-H	O…O	^2^ΔC(OD) ^a^
**2,4-di (B) ^b^**	1.2381	1.4455	1.4205	1.3333	0.9999	2.5980	0.302
**2,4-di (B)**	1.2379	1.4528	1.4255	1.3290	1.0000	2.5878	0.332
**2,3-dt (A)**	1.2319	1.4593	1.4184	1.3360	0.9909	2.6351	0.226
**5,6-dimethyl-2,3-dt (C)**	1.2364	1.4621	1.4066	1.3330	0.9944	2.5727	0.303

^a^ In ppm. ^b^ Letters refer to Table 2.

**Figure 8:**
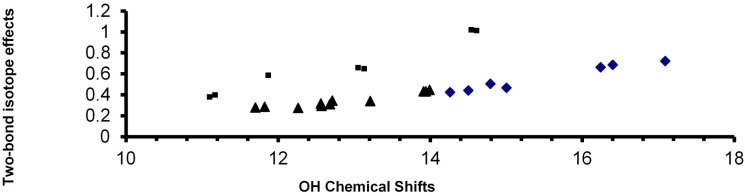
Plot of two-bond isotope effects *vs.* OH chemical shifts. Data taken from Refs. [26,27,28].

The reason for strong intramolecular hydrogen bonds lies in two factors, resonance assistance and in steric effects. The importance of resonance assistance is demonstrated above, steric effects are discussed below. Steric effects are important for IMHB as was seen in a number of *o*-hydroxyl acyl aromatics [27,28]. The effect of steric encumbrance can take two forms (i) the donor and the acceptor atoms stay in the plane of the double bond; (ii) one or both are twisted out of the double bond plane. An example of the former is shown in Figure 7C and of the latter in Figure 7D. The two-bond deuterium isotope effect in 7C has increased from 0.226 ppm as seen in Figure 7A to 0.303 ppm in the dimethyl derivative (7C) due to steric compression, so the O…O distance of course matters. This was also seen in a series of hydroxyl acyl benzenes [27]. The two types of steric effects can be distinguished as seen from Figure 8. Data for the twisted compounds fall high above the main correlation line [28].

**Figure 9:**
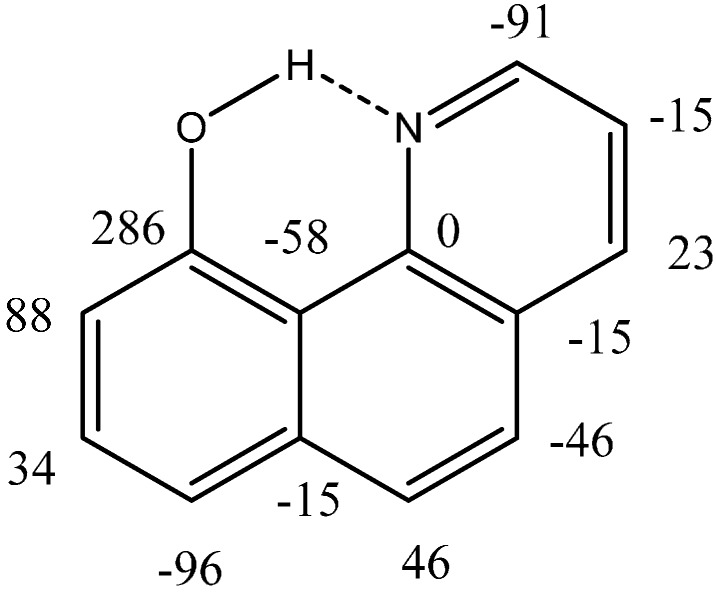
Deuterium isotope effects on ^13^C chemical shifts in 10-hydroxybenzo[h]quinolone.

An example of nitrogen being the acceptor is found in 10-hydroxybenzo[h]quinolines as seen in Figure 9. A series of compounds are investigated and a very good correlation is found between ^2^ΔC(OD) and δOH. However, the correlation is rather different from that of benzene derivatives due to the much stronger ring current effects of the 10-hydroxybenzo[h]quinolines compared to benzenes [29]. This influences the OH chemical shift, but not the isotope effects on chemical shifts, a good reason for using IECS rather that OH chemical shifts in estimating the strength of intramolecular hydrogen bonds.

As already mentioned do enaminocarbonyl compounds form intramolecular hydrogen bonds that can be monitored with IECS. Recently, enaminocarbonyl derivatives of Meldrum´s acid and tetronic acid have been investigated [30]. These compounds offer the possibility of comparing the NH…O=C-R system with that of the NH…O=C-OR system. Phenylenediamine derivatives of dehydroacetic acid [31] as well as salicyaldehyde-4-phenylthiosemicarbazone [32] and 5-acyl-3-methylrhodanines [33] are also investigated.

Systems with C=S groups of acceptors and OH groups as donors are found in *o*-hydroxythioacetophenones [34] and in hydroxyflavothiones [35]. Characteristics for these systems are larger two-bond deuterium isotope effects on ^13^C chemical shifts than in the corresponding ketones, large negative four-bond isotope effects on the C=S carbon and a presumably stronger hydrogen bond than in the corresponding ketone. In *o*-hydroxythioacetophenones isotope effects were also obtained in the solid state [34].

#### 2.1.2. Primary Isotope Effects

**Figure 10:**
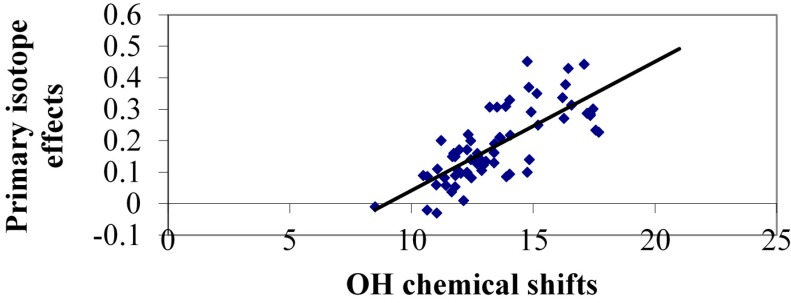
Plot of primary isotope effects, ^P^Δ(OD) *vs.* OH chemical shifts. Acceptors atoms are O, N or S. Data taken from Refs. [40,41].

As both primary deuterium isotope effects on ^1^H chemical shifts and OH chemical shifts represent the hydrogen bonded system it is interesting to plot those parameters against each other. From the plot of all primary deuterium isotope effects in “static” systems (Figure 10) it is seen that quite a spread exists. This is probably to some extent due to the variation of the OH chemical shift due to ring current effect *etc.* Some of the data points far from the correlation line are 2-nitrosonaphthol and others are 8-quinolinol N-oxides andcompounds in which the acceptor group is twisted out of the ring plane (see previously).

**Figure 11:**
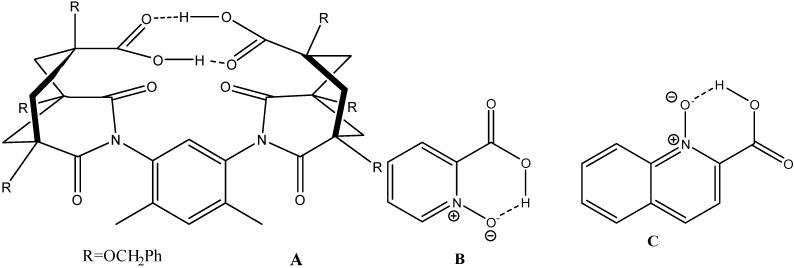
(**A**) *cis,cis*[4,6-dimethylbenzene-1,3-bis(1,5,7-tris(benzyloxymethyl)-2,4-dioxo-3-azabicyclo[3.3.1]non-3-yl-7-carboxylic acid)]; (**B**) picolinic acid N-oxide; (**C**) quinaldinic acid N-oxide.

**Figure 12:**
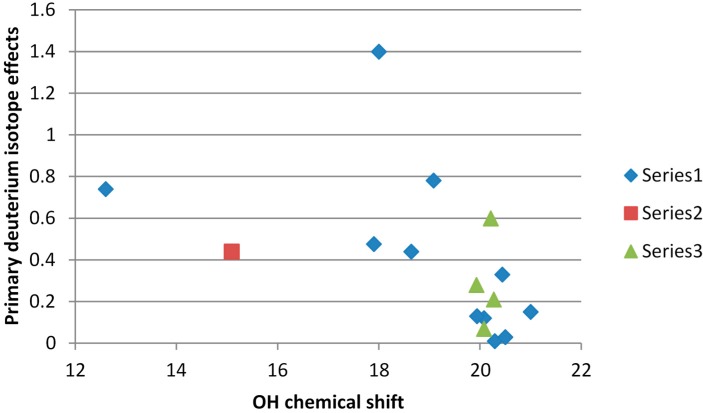
Plot of primary deuterium isotope effects for carboxylic acids *vs.* the OH chemical shift. Series 1 is; Data from Refs. [5,37,42,43] series 2 citrinin [44]; series 3 is data for mono anions of succinic acid [37].

If we look at carboxylic acids an interesting case is picolinic acid N-oxide (PANO) [5] and quinaldinic acid N-oxide [36]. (Figure 11) Both show rather large primary deuterium isotope effects for the acid proton. In case of PANO the effect vary very much in going from chloroform to acetonitrile as solvent. Guo *et al.* [37] investigated the intramolecular hydrogen bonds in monoanions of succinic acid and derivatives there of and established a double-well proton potential. Deuterium primary isotope effects of acid protons plotted *vs.* OH chemical shifts are given in Figure 12. The point for citrinin is clearly falling outside as this is tautomeric. However, the data for the succinic acid derivatives show no variation in the primary isotope effects despite the fact that the hydrogen bond potentials are different. The data points for the monoanion of the cage compounds (δOH, 18.0 ppm and ^P^ΔH(D), 1.4 ppm) [38] as well as the compound itself (δOH, 12.6 ppm and ^P^ΔH(D), 0.74 ppm) shown in Figure 11 are really very unusual. May the explanation is found in the author’s own statement “However, a bifurcated hydrogen bond with the acid placed between the oxygens cannot be excluded” or the effects should be compared to intermolecular hydrogen bonds as the flexibility is high [39].

### 2.2. Secondary Isotope Effects of Intramolecular Hydrogen Bonds without RAHB

**Figure 13:**
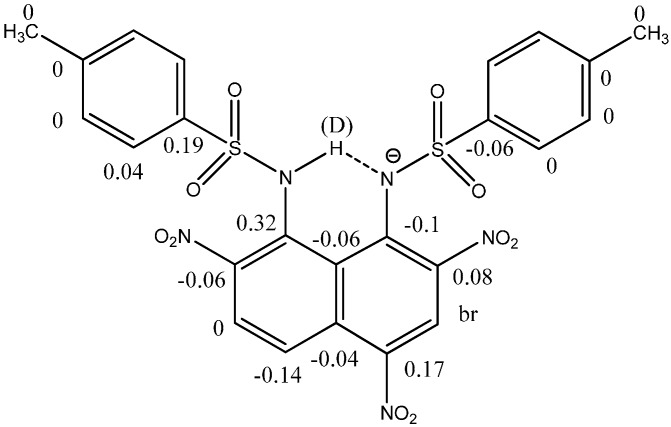
Deuterium isotope effects on ^13^C chemical shifts in 2,4,7-trinitro-1,8-bis(4-toluenesulphonamido)naphthalenes Taking the term intramolecular very far is to include a complex of carboxylic acids buried inside a cavity of a cavitand [46] and glycoluracil. However, some very interesting results are obtained. The acids are substituted benzoic aids and cinnamic acids. A nice correlation is obtained in a plot of deuterium isotope effects on the acid proton and the chemical shifts of that. Use of isotope effects avoids complications due to effects caused by interaction with the wall of the cavitand [47].

Isotope effects due to deuteriation of the NH proton of thioamides have been studied in detail and are reviewed in Ref. [2]. Likewise, hydrogen bonding in charged systems has been investigated. The pattern NH… N^−^ (see Figure 13) is found in the anion of 1,8-bis(4-toluenesulphonamido)naphthalenes and derivatives [44] (see Figure 13). These compounds may be “static” or tautomeric. The dinitro-derivative shown in Figure 13 is primarily on the form shown. The counter ion is DMANH+ and similar isotope effects for this moiety were observed as found previously [45]. 

### 2.3. Tautomerism

#### 2.3.1. Isotopic Perturbation of Equilibrium

**Figure 14:**
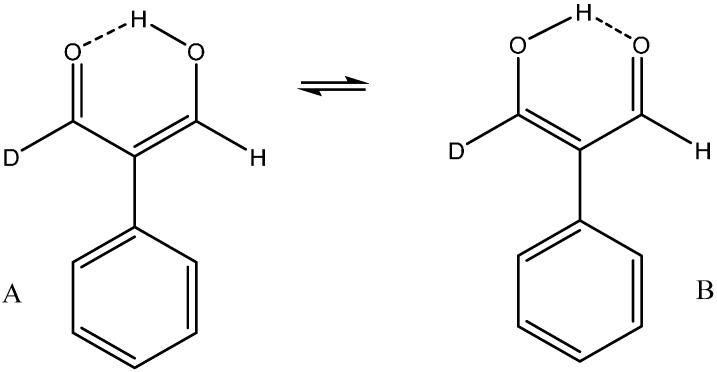
Isotopic perturbation of equilibrium.

One of the very elegant ways of disclosing tautomerism in symmetrical systems like β-diketones *etc.* is isotopic perturbation of equilibrium. This was demonstrated very neatly in 2-phenylmalonaldehyde [48,49,50]. For a symmetrical system the deuterium has to be introduced in such a manner that the degeneracy is lifted as demonstrated in Figure 14. Introduction of a deuterium at the chelate proton position will clearly not give rise to a shift in the equilibrium whereas as seen in Figure 14, whereas introduction at the aldehyde carbon will.

The perturbation is clearly caused by the vibrational difference between a deuterium at the aldehyde position and a deuterium at a double bond. The difference in the zero point energy for the two tautomers is 37 cm^–1^. This is resulting in a K = 1.2 in favor of the B-form as the stretching frequency of the C=C-H(D) bond is higher. The difference in chemical shift between the two relevant D’s in the two forms is ~2 ppm, so that the equilibrium primary isotope effects can be estimated to be ~0.1 ppm [50].

**Figure 15:**
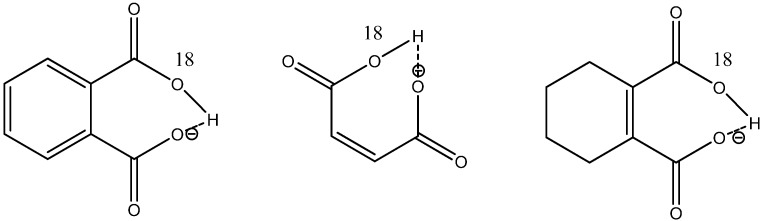
^18^O-labelled monoacids.

In non-symmetrical systems measurement of deuterium isotope effects on e.g., ^13^C or ^15^N chemical shifts will result in an s-shaped dependence on mole fraction [51,52] as well as in isotope tope effects far from the center of deuteriation. One of the hotly debated issues is the symmetry of the systems shown in Figure 12 and related to that, low barrier hydrogen bonds. This subject has been reviewed thoroughly by Perrin [48,49,53]. One of the main tools in these studies has been isotopic perturbation of equilibrium caused by substitution with ^18^O (see Figure 15). The idea is that as the isotope effects observed are larger than the intrinsic effects normally found, an equilibrium has to be at play and therefore a double well potential.

Recently, Bogle and Singleton [54] suggested that large intrinsic isotope effects can occur in such systems. They based their argument on desymmetrization. In order to clarify the situation Perrin and Burke [41] have measured the ^18^O isotope effects on ^13^C chemical shifts in ^18^O labelled cyclohexene-1,2-dicarboxylate (Figure 15) at different temperatures. They found that the isotope effect at the ipso carbon increased when they lowered the temperature. This they took as support of a tautomeric equilibrium. Using ^18^O isotope effects on chemical shifts combined with solvent effects, they reached the same conclusion for the mono-anion of racemic-α,α'-di*-tert*-butyl succinate [55,56] and of difluoromaleate monoanion [57].

**Figure 16:**
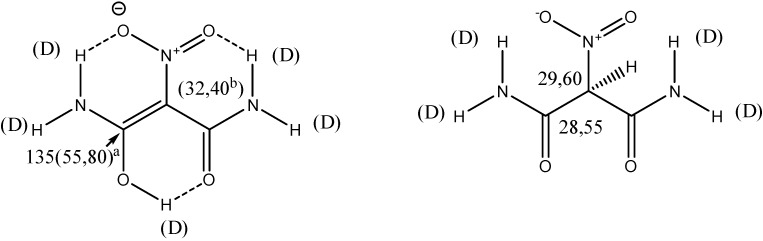
Deuterium isotope effects of nitromalonamide.

Enolised double bonds are not very common. An example is nitromalonamide (Figure 16) [42]. Nitromalonamide is a model system for tetracyclines.

#### 2.3.2. Primary Isotope Effects

##### Symmetrical Systems

**Figure 17:**
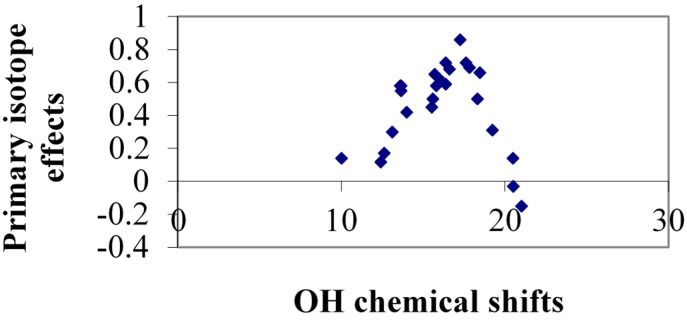
Plot of primary isotope effects *vs.* OH chemical shifts for symmetrical systems based on data from Refs. [39,59].

Primary isotope effects should in this context first be discussed for deuteriation in which the symmetry is not broken. The primary deuterium isotope effects of the chelated protons can with advantage be correlated with the OH chemical shift. A bell shaped curve is found as seen in Figure 17. This was earlier suggested by Cassidy, Liu and Fry [40,58] as well as by Sobczyk *et al.* [59], but based on fewer data. The bell shaped curve is a consequence of the change from weak, to strong, to short and strong hydrogen bonds (SSHB) and the concomitant change in the hydrogen bond potential from a double potential well to an single well potential. In the latter case a negative primary isotope effect is observed. A certain spread is to be expected as effects such as ring currents will influence the OH chemical shift, but ring current effects will cancel out for the isotope effects. A risk dealing with hydrogen bonded protons is always that exchange may lead to erroneous results. 

One of the difficult matters to resolve is the existence of single hydrogen bond potentials and to define unambiguously the existence and what criteria to use. Perrin and Nielson [60] have investigated primary deuterium in a series of mono anions of phthalic acid, 1,2-cyclopentene dicarboxylic acid, 3,4-furandicarboxylic acid and 3,4,5,6-tetrahydrophthalic acid in organic solvents and at different temperatures. The measurement of ^p^ΔH(D) were supplemented by also studying ^18^O isotope effects on ^13^C chemical shifts (see above). For the mono anions of 1,2-cyclopentene dicarboxylic acid, 3,4-furandicarboxylic acid and 3,4,5,6-tetrahydrophthalic acid the primary isotope effects are positive or zero in line with the ^18^O results and with prior finds for 3,4-furandicarboxylic acid [40] showing a strong, asymmetric hydrogen bond. For the phthalate the primary isotope effects can be either positive or negative depending on the solution, showing that recording of these small primary isotope effects call for caution.

The first part of the curve of Figure 17 is very similar to that seen for the “static” cases of Figure 10 underlining that for symmetrical systems in which the degeneracy is not lifted, the effects are intrinsic.

##### Non-Symmetrical Systems

**Figure 18:**
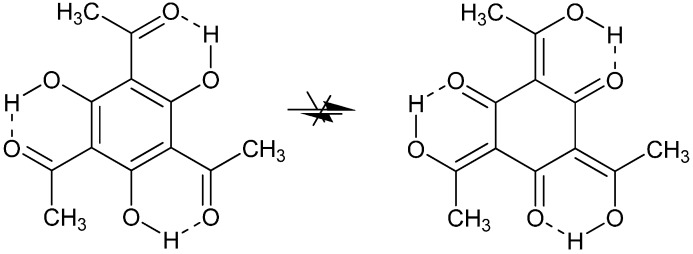
l,3,5-trihydroxy-2,4,6-triacetylbenzene.

Deuterium isotope effects on ^13^C chemical shifts have been used to demonstrate that l,3,5-trihydroxy-2,4,6-triacetylbenzene is not taking part in tautomerism (see Figure 18) [61] although this had been claimed based on theoretical calculations [62].

**Figure 19:**
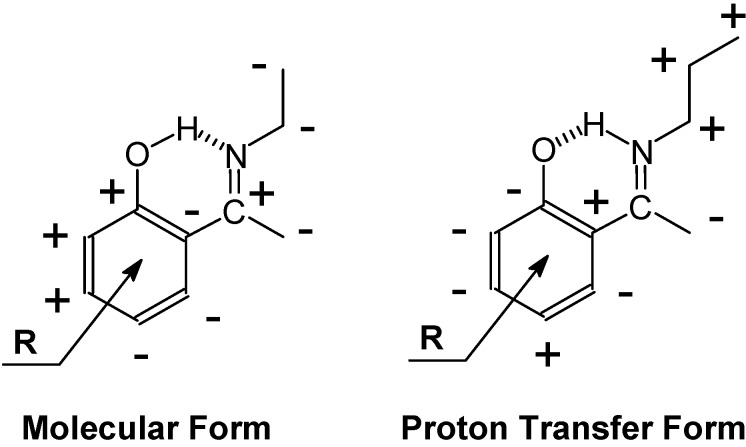
Deuterium isotope effect on ^13^C chemical shift sign patterns of *o*-hydroxy Schiff bases.

Tautomeric equilibria have been established in many *o*-hydroxy Schiff bases using IECS. Deuterium isotope effects on chemical shifts have been shown to be especially powerful due to the very large chemical shift differences between the ^15^N chemical shifts in the OH and the NH forms (also referred to as molecular and proton transferred form) (See Figure 2) (100–140 ppm) [52]. A very nicely S-shaped dependence is seen between ^1^Δ^15^N(D) and the mole fraction [63]. Also deuterium isotope effects on ^13^C chemical shifts have been used in many cases and sign patterns can distinguish between the two forms [64] (Figure 19). Furthermore, the observation of long range effects clearly differentiates between “static” and tautomeric cases. That equilibrium is at play may also be demonstrated by plotting isotope effects for two different carbons at different temperatures against each other and observing a straight line [65]. The intrinsic and the equilibrium part of the isotope effects can be separated. This is demonstrated in two different ways by Filarowski *et al.* [63] and by Limbach *et al.* [11]. Filarowski *et al.* used the equations described earlier combined with calculation of nuclear shieldings and isotope effects Limbach *et al.* used the geometric approach described in Ref. [66] combined with an equilibrium approach. One-bond deuterium isotope effect on ^15^N chemical shifts are plotted *vs.* the ^15^N chemical shifts for Schiff bases. The fit to the predicted solid lines is rather poor [11]. *o*-Hydroxynaphtalene Schiff bases of methyl amine are investigated with respect to solvent effects. The 1-OH derivatives are more sensitive to solvent polarity [67].

Schiff bases of pyridoxal-5'-phosphate is a cofactor in many enzyme reactions. Isotope effects on ^15^N chemical shifts have been measure in ^15^N-labelled material. Protonation of the pyridine nitrogen play a role for the position of the Schiff base tautomeric equilibrium, which is shifted towards the NH-form [68]. That charge may play a role was also previously demonstrated in Schiff bases of salicylaldehydes [69] or 2-hydroxynaphtaldehyde [70] with amino acids. In amino acid ionic liquids supported Schiff bases the presence of the COO^−^ group stabilizes the proton transferred form [71].

Tautomeric Schiff bases with relay have also been studied and anti-cooperativity has been established [72]. A coupled system is also found in 2,7-diacetyl-1,8-dihydroxy-3,6-dimethylnapthalene both in the liquid and solid state [73].

Deuterium isotope effects on chemical shifts have been studied to a great detail in both β-diketones and β-thioxoketones in solution [1,51]. Recently, also deuterium IECS of thiodibenzoylmethane has been reported in the solid state. In this case the effects were assumed to be intrinsic [34]. However, deuterium IECS in the solid state spectra of pyridoyl benzoyl β-diketones reveal a tautomeric equilibrium in the solid state [74].

**Figure 20:**
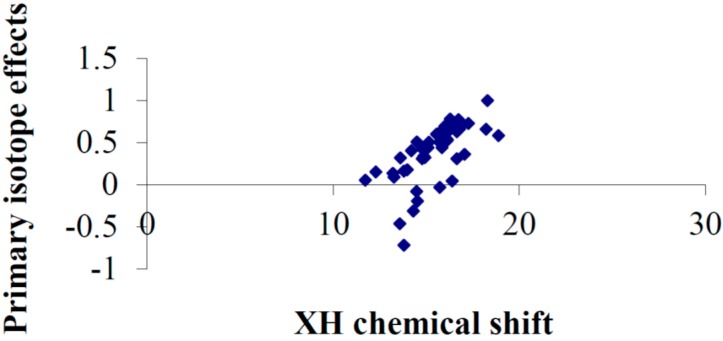
Plot of primary isotope effects *vs.* XH, X being O, N or S, chemical shifts for tautomeric systems. Data primarily from Ref. [39,76] and those references mentioned above.

A plot including tautomeric compounds like phleichrome [75], isophleichrome [75], 2-pivaloylindane-1,3-dione [76], 5-acetyl-1,3-dimethyl-2,4,6-trioxo-1,3-diazane, 5-acetyl-2,2-dimethyl-4,6-dioxo-1,3-dioxane [76] and oxo-(2-oxo-cyclohexyl)-acetic acid ethyl ester [77] but also β-thioxoketones [78] show that the primary isotope effects may be large and negative (Figure 20). This is typical for a tautomeric system with the two different heavy atoms like O and S as found in β-thioxoketones.

**Figure 21:**
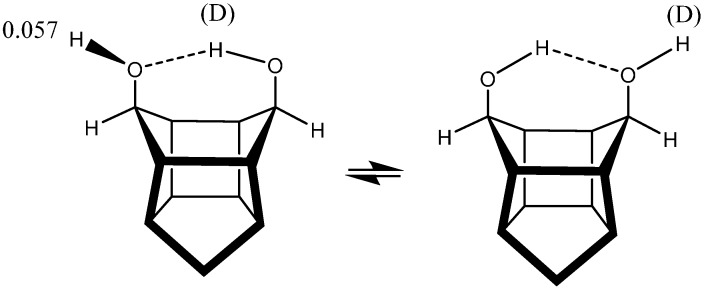
Deuterium isotope effects on ^1^H chemical shifts in cage compound.

Equilibrium isotope effects are also found in the cage compound (*endo,endo*)-pentacyclo[5.4.0.0^2,6^.0^3,10^.0^5,9^]undecane-8,11-diol [79] Figure 21. They are slightly different in Ref. [12] as compared to Ref. [79] because of different amount of water present. The equilibrium isotope effects are discussed in Ref. [79] in relationship to carbohydrates.

## 3. Summary

Isotope effects on chemical shifts both primary and secondary are very good tools in the study of intramolecular hydrogen bonds as they are intimately linked to zero bond energies and hence to hydrogen bond potentials. Resonance assisted hydrogen bonding together with steric compression is demonstrated to play a major role in the strength of intramolecular hydrogen bonds. IECS are preferable to XH chemical shifts in such studies as external effects like solvents, cage walls *etc.* do not play a role. IECS are likewise very useful in the study of tautomeric systems both symmetric and asymmetric. In the former case isotope perturbation of equilibrium is the preferred tool and combined with temperature experiments they have been the key tool to prove a double bond potential in such systems like phthalic acid mono anion.
